# Human amnion-derived mesenchymal stem cells improved the reproductive function of age-related diminished ovarian reserve in mice through Ampk/FoxO3a signaling pathway

**DOI:** 10.1186/s13287-021-02382-x

**Published:** 2021-06-02

**Authors:** Hanwen Liu, Chunyan Jiang, Boya La, Meng Cao, Song Ning, Jing Zhou, Zhengjie Yan, Chuyu Li, Yugui Cui, Xiang Ma, Meilian Wang, Li Chen, Youjia Yu, Feng Chen, Yuexin Zhang, Huimin Wu, Jiayin Liu, Lianju Qin

**Affiliations:** 1 State Key Laboratory of Reproductive Medicine, Center of Clinical Reproductive Medicine, First Affiliated Hospital, Nanjing Medical University, Nanjing, 210029 China; 2 Department of Obstetrics, First Affiliated Hospital, Nanjing Medical University, Nanjing, 210029 China; 3 Department of Forensic Medicine, Nanjing Medical University, Nanjing, 211166 Jiangsu China; 4 Key Laboratory of Targeted Intervention of Cardiovascular Disease, Collaborative Innovation Center for Cardiovascular Disease Translational Medicine, Nanjing Medical University, Nanjing, 211166 Jiangsu China

**Keywords:** Age-related diminished ovarian reserve, Human amnion-derived mesenchymal stem cells, Granulosa and stromal cells, AMPK/FoxO3a signaling pathway, Oxidation resistance, Mitophagy

## Abstract

**Background:**

Age-related diminished ovarian reserve (AR-DOR) reduced the quality of oocytes, resulting in decreased female fertility. Aging is tightly related to abnormal distribution and function of mitochondria, while mitophagy is a major process to maintain normal quality and quantity of mitochondria in cells, especially in oocytes which containing a large number of mitochondria to meet the demand of energy production during oocyte maturation and subsequent embryonic development. Ampk/FoxO3a signaling is crucial in the regulation of mitophagy. It is reported mesenchymal stem cells (MSCs) can improve ovarian function. Here we aim to explore if human amnion-derived mesenchymal stem cells (hAMSCs) are effective in improving ovarian function in AR-DOR mice and whether Ampk/FoxO3a signaling is involved.

**Methods:**

The AR-DOR model mice were established by 32-week-old mice with 3–8 litters, significantly low serum sex hormone levels and follicle counts. The old mice were divided into 5 treatment groups: normal saline (NS, control), 1% human serum albumin (HSA, resolver), low dose (LD, 5.0 × 10^6^cells/kg), middle dose (MD, 7.5 × 10^6^cells/kg), and high dose (HD, 10.0 × 10^6^cells/kg). The prepared hAMSCs were injected through tail vein. Serum sex hormone level, follicle counts, fertilization rate, gestation rate, little size, apoptosis of granulosa and stromal cells, expression level of Sod2, Ampk, and ratio of phosphorylated FoxO3a to total FoxO3a in ovaries were examined.

**Results:**

Our results show that after hAMSC transplantation, the ovarian function in AR-DOR mice was significantly improved, meanwhile the apoptosis of granulosa and stromal cells in the ovaries was significantly repressed, the expression level of Ampk and the ratio of phosphorylated FoxO3a to total FoxO3a both were significantly increased, meanwhile increased Sod2 expression was also observed.

**Conclusion:**

Our results demonstrate hAMSC transplantation via tail-injection can improve ovarian function of AR-DOR mice through Ampk/FoxO3a signaling pathway.

**Supplementary Information:**

The online version contains supplementary material available at 10.1186/s13287-021-02382-x.

## Background

Diminished ovarian reserve (DOR) in female refers to the decline in the quality and quantity of oocytes in the ovaries, accompanied by menstrual abnormalities, ovulation disorders, infertility, and organ and systemic decline related to hypoestrogen [1–3]. According to the European Association for Assisted Human Reproduction bologna Conference standards, the diagnostic criteria of DOR are anti-Mullerian hormone (AMH) 0.5–1.1 μg/L and antral follicle counting (AFC) < 5–7. Currently, DOR accounts for about 10% of infertile women [1]. The causes of DOR are diverse. It is named age-related DOR (AR-DOR) when age acts as the independent factor. According to current consensus, the age over 35 years old of women who have fertility requirements is defined as advanced reproductive age, which can lead to AR-DOR, showing physiological ovarian degeneration [4].

Recently, AR-DOR patients are gradually increasing due to the increasing of female reproductive age [5]. However, traditional artificial assisted reproduction (ART) treatment with increased ovarian stimulation doses has been proven not to increase the live birth rate of AR-DOR women [6], and other treatments (including hormone regulation, antioxidant application, growth hormone addition) neither show satisfied clinical outcomes [7–9]. Oocyte mitochondrial transplantation might be an effective treatment for mitochondria dysfunction which is the typical symptom of AR-DOR [6], while there are still technical, ethical, and regulatory limitations in the technology [10, 11].

It is necessary to explore an effective treatment for AR-DOR patients. Accumulation of reactive oxygen species (ROS) in the ovary is the basic pathological change and the main cause of low quality of oocytes in AR-DOR patients [12, 13]. The excess ROS not only can increase apoptosis of granulosa and stromal cells in ovary, indirectly impeding the development and maturation of oocytes, but also harm the function of mitochondria in oocyte itself, directly restraining the maturation of oocytes [14, 15]. Thus, treatments effective in resisting ROS may promise to improve the quality of oocytes in AR-DOR patients [16]. In recent years, investigators have disclosed that transplantation of mesenchymal stem cells (MSCs) or their secretions, including placenta-derived mesenchymal stem cells (PD-MSCs) [17], human umbilical cord mesenchymal stem cells (UCMSCs) [18, 19], adipose-derived stem cells (ADSCs) [20, 21], or ADSC-conditioned medium [22], can all significantly improve ovarian function in premature ovarian failure (POF) or primary ovarian insufficient (POI) models. Furthermore, it is reported that transplantation of UCMSC functions on POI patient [23]. Further explorations demonstrate that MSCs improved ovarian function through oxidation resistance in which PI3K-FoxO3a or SIRT signal pathway is involved [24, 25].

Human amnion-derived mesenchymal stem cell (hAMSC) is a type of multipotent adult stem cell isolated from the amnion which is usually treated as medical waste. Similar to other MSCs, hAMSCs possess the ability of self-renewal, differentiation, homing, and secretion of growth factors [26, 27]. So far, hAMSCs or their secretions have been showed effective in rescuing ovarian function in chemical-induced POI or POF model when directly injected into ovarian tissue via improving the follicular microenvironment and SIRT4 signaling may be involved [28–30]. However, the therapeutic potential, the dose, and the mechanisms of hAMSCs on the ovary function in AR-DOR model have not yet been explored.

In present study, a practical AR-DOR mouse model was first established, clinical-grade hAMSCs were prepared and transplanted by tail intravenous injection, cells homing was detected, ovary function was evaluated, and oxidation resistance and mitophagy-related signaling pathway were analyzed. Our results demonstrate that middle (7.5 × 10^6^ cells/kg) and high (10.0 × 10^6^ cells/kg) dose of hAMSC transplantation improved the ovarian function by Ampk/FoxO3a signaling mediated mitophagy in oocytes in AR-DOR mice.

## Methods

### Isolation, characterization, and preparation of hAMSCs

The application of human amnions in this study was approved by the institutional ethics committee of First Affiliated Hospital, Nanjing Medical University. Human placentas were collected from a healthy, normal term pregnant woman who underwent cesarean section due to simple hip circumference factors after written and informed consent was signed. Amnion-derived mesenchymal stem cells (hAMSCs) were isolated according to previous reports [31]. Briefly, amniotic membrane was sequentially removed from the placental chorionic layer under aseptic conditions, cleaned to remove the blood cells by DPBS (Gibco, A12856-01), contained Penicillin-Streptomycin (Gibco, 15140), cut into small pieces, and immersed in TrypLE (Gibco, 12605-010) at 37 °C for 30 min with vigorous shaking. The partly digested tissues were transferred into new tubes, washed with DPBS, immersed again in fresh TrypLE at 37 °C with vigorously shaking until digested completely. Single cells were collected by centrifuge at 1500 rpm for 5 min and plated in PLT medium which is composed of α-MEM medium (Gibco, 12571-063), 5% UltraGRO-hPL (Helios Bioscience, HPCPLCRL50), 1% L-Glutamine (Gibco, 25030), and 0.03% Heparin Sodium (Changzhou Qianhong Bio-pharma CO.Ltd., H32022088), incubated at 37 °C and 5% CO_2_. Culture cells were trypsinized and passaged when 80–90% confluence is observed. The cell line AMSC10 of human amnion mesenchymal stem cells (hAMSCs) was established, and its identity, genetic safety, biological safety, toxicology, tumorigenicity, pluripotency, biological activity, and safe dose were completely assessed and all tested items were up to standard (submitted and under review data).

In the study, the fifth-passage hAMSCs were prepared and applied in all of transplantation experiments. Release inspection on the expression of MSC-specific surface markers, concentration of secreting cytokines in cell culture supernatant, cell viability, bacteria contamination, and mycoplasma infection cells were carried out before hAMSC transplantation. MSC-specific surface markers were detected by flow cytometry. The hAMSCs were harvested, washed, and resuspended with PBS. Cells were counted and diluted to 2 × 10^6^ cells/100 μl. Subsequently, cells were incubated with monoclonal phycoerythrin-conjugated antibodies for human CD44 (BD, 555479), CD90 (BD, 555596), CD73 (BD, 550257), CD105 (eBioscience, 2-1057-42), CD11b (BD, 555388), CD19 (BD, 555413), CD34 (BD, 555822), CD45 (BD, 555483), and HLA-DP/DQ/DR (BD, 562008). Appropriate isotype-matched antibodies were used as negative controls. The data from 10,000 viable cells were acquired with a flow cytometer (Beckmen, NAVIOS) and analyzed using the Kaluza software (BECKMAN COULTER: version 1.3). Cell viability were calculated by cell counting using a blood cell counting chamber (Reichert Bright-line, Cat.1483), bacteria contamination were assessed by endotoxin detection kit (Zhanjiang Bokang Marine Biological Co., Ltd, China) according to manufacturer’s instructions, and mycoplasma infection were assessed by real-time fluorescence quantitative polymerase chain reaction with specific primers (Forward primer: GGGAGCAAACAGGATTAGATACCCT and reverse primer: TGCACCATCTGTCACTCTGTTAACCTC) on an Applied Biosystem® CO.Ltd. machine (QuantStudio^TM^ 7 Flex Real-Time PCR System). Qualified hAMSCs were suspended in 1% human serum albumin (HSA), filled with 300 μl/tube containing a defined number of cells according to different doses.

### Isolation and culture of HFFs

The application of human foreskin in this study was approved by the institutional ethics committee of First Affiliated Hospital, Nanjing Medical University. The human foreskin fibroblasts (HFFs) were isolated from the 7-year-old boy’s foreskin. Foreskin was cleaned to remove the blood cells by DPBS (Gibco, A12856-01), contained Penicillin-Streptomycin (Gibco, 15140), cut into small pieces (2 mm), and then plated in PLT medium. Growing cells were harvested by TrypLE (Gibco, 12605-010) digestion and replated to establish the HFF cell line. In this study, the fifth-passage cells were applied in transplantation experiments (HFFs group, 7.5 × 10^6^cells/kg).

### Analysis for the pluripotency of hAMSCs in vitro

Cells that reached 80–90% confluence were digested with TrypLe and washed with DPBS.

For adipocyte and osteoblast differentiation, hAMSCs were resuspended by PLT medium seeded on 24-well culture plates (6 × 10^4^ cells in each well) and cultured for 48 h. PLT medium was then changed to StemPro Adipogenesis Differentiation Kit (Gibco, A1007001) or StemPro Osteogenesis Differentiation Kit (Gibco, A1007201) with 21 days culture. The adipogenesis differentiation group was stained with Oil Red O for lipid droplets, while the osteogenic differentiation group was stained with Alizarin red (Sigma-Aldrich, A5533) for mineralized nodules.

For chondrocyte differentiation, hAMSCs were resuspended by PLT medium, seeded on U-bottomed 96-well plates (1 × 10^5^/200ul in each well) and cultured for 48 h. PLT medium was then changed to StemPro Chondrogenesis Differentiation Kit (Gibco, A1007101) with 21 days culture. The cells were fixed with 4% paraformaldehyde (PFA), embedded in paraffin, sectioned, and deparaffinized. They were then stained with Alcian blue (Sigma-Aldrich, 66011) for 30 min and washed by 3% glacial acetic acid. The cells were photographed by a microscope (Nikon, Eclipse Ni-U).

### Animals

The application and handle of specific pathogen-free grade C57BL/6 mice was approved by the Animal Ethics Committee of Nanjing Medical University. Eight-week-old male and female and twenty-eight-week-old female C57BL/6 mice were purchased from Beijing Vital River Laboratory Animal Technology Co., Ltd. The mice were housed in the SPF-class animal room of Nanjing Medical University. The mice were grouped in 5 mice per cage, were free to eat and drink, and were exposed to a day/night discontinuous illumination (12 h:12 h). The food, litter, water, and cages used for feeding were strictly disinfected and sterilized.

### Establishment of age-related diminished ovarian reserve (AR-DOR) mouse model

It is reported 32-week-old C57BL/6 mice are equivalent to about 35 years old of human [32], whose fertility have been proved gradually decreasing [33]. Thus the 32-week-old female mice were selected to establish the age-related DOR (AR-DOR) model. The 8-week-old female mice were set as control. Total 200 female mice at 27-week age were purchased and screened firstly by littler size after one time breeding. Then 118-day-old mice (32-week-old) with 3–8 litters were selected to further analysis. Venous blood samples were collected from control mice and old mice. Levels of anti-Mullerian hormone (AMH), estrogen (E2), and follicle-stimulating hormone (FSH) in serum were accessed. Furthermore, old female mice were humanely sacrificed and follicles at different development stages in ovaries were count and analyzed. The old female mice had significantly less litter size, lower levels of AMH and E2 in serum, and lower numbers of secondary and antral follicles, indicating the AR-DOR model was successfully established (Fig. 2).

### Establishment of premature ovarian failure (POF) mouse model

Eight-week-old female C57BL/6 mice were exposed to whole-body X-ray (4 Gy) to establish a premature ovarian failure (POF) model. The untreated 8-week-old female mice were set as control. Serum sample were collected in control mice and radiated mice to analyze the level of AMH, E2, and FSH. In addition, the follicles at different development stages and ultrastructure in ovaries were analyzed. The radiated mice had a significantly higher level of FSH, lower level of AMH and E2 in serum, and lower numbers of primordial, secondary, and antral follicles, indicating the POF model was successfully established (Figure S4).

### Cell transplantation and mice treatment on endpoints

#### Assess effects of hAMSCs on ovarian function of DOR mice

The safe dose of hAMSCs via tail vein injection in mouse is below 5.0 × 10^7^cells/kg body weight according to our exploration (submitted and under review data). The AR-DOR model mice were randomly assigned into 5 groups (22 mice/group): the normal saline group (NS), the 1% human serum albumin group (HSA), the low-dose group (LD, 5.0 × 10^6^cells/kg), the medium-dose group (MD, 7.5 × 10^6^cells/kg), and the high-douse group (HD, 1.0 × 10^7^cells/kg). Each mouse was slowly injected with 300 μl of the corresponding preparation through the tail intravenous for total 3 times at a 4-day interval [30, 34]. Seven days after the last injection, venous blood was collected from 8 mice of each group to perform hormone assay, and then humanely sacrificed to carry out follicles and oocyte counting, 2-cell and blastocyst formation, apoptosis analysis, histology, and immunofluorescence assay, and 6 mice from each group were humanely sacrificed to collect ovary to perform western blot assay. About 2 weeks after the last injection, the left mice from each group were humanely sacrificed to perform analysis on gestation rate and number of live fetus.

#### Assess immune reactions of transplanted hAMSCs on ovarian function of DOR mice

DOR mice were divided into three groups: HSA group (7 mice), human foreskin fibroblasts (HFFs) group (5 mice, 7.5 × 10^6^cells/kg body weight), and hAMSC group (7 mice, 7.5 × 10^6^cells/kg body weight). The ovarian function of transplanted mice were assessed after 3 times of injection (at a 4-day interval, 300 μl/mouse/time) and followed by 7 days of recovery.

#### Assess effects of 300 μl injection volume on heart function of DOR mice

DOR mice were divided into three groups (8 mice/group): no injection (NI) group, HSA group, and hAMSC group (7.5 × 10^6^cells/kg body weight). The echocardiography and pathological sections of heart tissue were analyzed after 3 times of injection (at a 4-day interval, 300 μl/mouse/time) and followed by 7 days of recovery.

### Enzyme-linked immunosorbent assay (ELISA)

The mice (36-week-old) on the day 7 of the last hAMSC transplantation were injected intraperitoneally with anesthetic until their limb muscles became weak. About 1 ml/mouse blood was taken from the eye posterior orbital venous plexus. The obtained blood was left at room temperature for 30 min, then serum was collected following centrifugation at 3000 rpm for 15 min and stored at − 80 °C before hormone analysis. The levels of E2, FSH, LH, and AMH were measured according to the manufacturer’s guide of ELISA kit (Jining Shiye, A05182, A05021, N05078, N04308) on a full-wavelength microplate reader (Thermo Scientific, Multiskan GO).

HAMSC culture supernatant was collected and stored at − 80 °C for analysis. The secreting cytokine level of interleukin-6 (IL-6), fibroblast growth factor 7 (FGF7), and angiopoietin-1 (ANG-1) were measured according to the manufacturer’s guide of ELISA kit (Multi Sciences, 70-EK106/2, 70-EK1262, 70-EK1122) on a full-wavelength microplate reader (Thermo Scientific, Multiskan GO).

### Oocyte counting, in vitro fertilization, and embryo culture

Oocytes from each group (8 mice/group) were harvested, counted, and then performed in vitro fertilization and in vitro embryo culture to evaluate the effects of hAMSC transplantation on the egg quality. A sperm suspension was prepared at least 1 h before fertilization. In brief, 10-week-old C57BL/6 males were sacrificed by cervical dislocation. Epididymis was dissected and cut by a single-use needle. Spermatozoa were overflowed into a drop of Human Tubal Fluid (HTF: EasyCheck, M1130) medium covered with mineral oil (Sigma, M8410) and incubated at 37 °C for 60 min (for capacitation). Then the female mice injected different preparation were humanely sacrificed by cervical dislocation. Oviducts were dissected and cut open the fallopian tube where the enlarged and bright ampulla was picked by a single-use needle and transferred in M2 media (Sigma, M7167). In vivo-matured oocytes (within cumulus follicular complex, COCs) were incubated with spermatozoa for 5 h. Zygotes were washed and cultured in KSOM (EasyCheck, M1430) embryo culture medium under mineral oil in groups of 15–20 embryos per drop (30uL). The number of zygotes after fertilization was recorded as the number of eggs obtained in mice. Embryos were cultured at 37 °C with 5% CO_2_. The proportion reaching the 2-cell (24–30 h after fertilization) and blastocyst (96–100 h after fertilization) stages were recorded.

### Ovarian morphology analysis and follicle counting

Ovaries from each group (8 mice/group, 36-week-old) were collected at a week after the last hAMSC transplantation, fixed in 4% paraformaldehyde at room temperature overnight, then dehydrated, embedded in paraffin, sliced into 5-μm serial sections, and stained with hematoxylin and eosin (H&E) according to standard protocol. The follicles containing oocytes with a visible nucleus were counted in every fifth section of the entire ovary and were scored as primordial, primary, secondary, or antral follicles based on their morphological appearances as described previously [35]. Briefly, primordial follicles were classified as an oocyte surrounded by one layer of flattened granulosa cells, primary follicles were classified as an oocyte surrounded by one layer of cuboidal granulosa cells, secondary follicles were classified as an oocyte surrounded by more than one layer of cuboidal granulosa cells with no visible antrum, and antral follicle were classified as an oocyte surrounded by multiple layers of cuboidal granulosa cells and containing one or more antral spaces.

### Gestation rate and live fetus number assay

The female and male mice were combined in a 2:1 cage for 2 weeks beginning from day 7 of the last injection, and the semen plug picked up was marked as E0.5. The female mice were injected with 0.4% trypan blue solution (Gibco, 15250061) into the tail vein in E5.5, and the uterus was removed by abdominal surgery. The number of blue bands around uterine horns was counted [36], and gestation rate was calculated by the formula: number of pregnant females/number of inseminated females [37].

### Apoptosis assay

A TUNEL apoptosis assay kit was used to detect ovarian cell apoptosis in each group at a week after the last hAMSC transplantation according to the manufacturer’s instructions (Beyotime, C1098). Nuclei of apoptotic cells were stained dark brown. Sections were observed and imaged using an optical microscope. The apoptotic cells on sections were counted using a double-blind method and analyzed by two technicians.

### Histology and immunofluorescence

Immunofluorescence experiments were carried out to trace hAMSC homing and differentiation and evaluated the expression level of phosphorylated FoxO3a in ovary after cell transplantation. Paraffin-embedded sections of ovaries were dewaxed, and heat-mediated antigen retrieval was performed by microwaving for 20 min in 10 mM sodium citrate (pH 6.0) (Beyotime, P0083). The sections were cooled for 15 min, washed in deionized water, rinsed in PBS, incubated in 5% goat serum for 60 min, then incubated overnight at 4 °C with primary antibodies of anti-human STEM121 antibody (1:100, TAKARA, Y40410), mouse anti-human CD73 antibody (1:100, Abcam, ab133582), and rabbit anti-FoxO3a antibody (1:100, CST, 12829) followed by incubation with secondary antibodies of CoraLite488 conjugated Goat Anti-mouse IgG (H + L) (1:100, Invitrogen, A21202) and Goat anti-rabbit IgG (H + L)(1:100, Abcam, ab150078) for 1 h at room temperature. DAPI (Beyotime, C1006) was used for DNA counter staining. The signals were acquired by performing the same immunostaining procedure and setting up the same parameters with a confocal microscope (Nikon, ECLIPSE Ti).

### Western blot

The ovaries from NS, HSA, and hAMSC groups were pooled separately in SDS-PAGE protein loading buffer (Beyotime, P0015) and RIPA Lysis and Extraction Buffer (Thermo Fisher, 89900) with protease and phosphatase inhibitor Cocktail (Thermo Fisher, 78443). Then samples were denatured at 95 °C for 10 min and stored at − 80 °C. After thawing, they are loaded on a 10% SDS-PAGE and blotted on polyvinyl fluoride (PVDF) membranes (Bio-Rad, 1620177). Non-specific binding sites were blocked for 1 h at 37 °C with 5% no-fat dry milk (BD, 2321000) in Tris-buffered saline containing 0.05% Tween 20 (TBS-T). Membranes were incubated with polyclonal rabbit anti-Sod1 (1:1000, Abcam, ab13498) , rabbit anti-FoxO3a (1:1000, CST, 12829), rabbit anti-p-FoxO3a (1:1000, CST, 9466), rabbit anti-Ampk (1:1000, Abcam, ab133448), rabbit anti-FSHR (1:1000, Proteintech, 22665-1-AP), rabbit anti-Cyp17a1(1:1000, ABGENT, AP7879c), and rabbit anti-GAPDH (1:5000, Proteintech, 10494-1-AP) overnight at 4 °C, followed by incubation with horseradish peroxidase (HRP) conjugated anti-rabbit secondary antibody (1:1000, Abcam,ab6721) for 1 h at room temperature. After washing, specific immunoreactive complexes were detected by ECL kit (Thermo Fisher, 32209). The bands were normalized for Gapdh using ImageJ software (NIH, Bethesda, MD, USA), and values were given as relative units. The experiment was performed in triplicate.

### Echocardiography

Mice were anesthetized with 1.5% isoflurane and imaged in the supine position using a Vevo 3100 Imaging System with a 30-MHz linear probe (Visualsonics, Canada). Core temperature was maintained at 37 °C. A standard 2D echocardiographic study was initially performed in the parasternal long-axis view for assessment of LV dimensions and systolic function. Image depth, width, and gain settings were used to optimize image quality.

Two-dimensional echocardiography was used to visualize left ventricular (LV) wall segment’s motion. Thickness of LV anterior and posterior walls at end cardiac diastole (LVAWd, LVPWd) and LV dimensions at end cardiac diastole and systole (LVDd, LVDs) were measured by M-mode echocardiography (M-mode). LV fractional shortening (FS) and LV volumes at end cardiac diastole and systole (LVVd, LVVs) were determined, and the LV ejection fraction was computed from the Vivid 7 system software: FS = (LVDd-VDs)/LVDd× 100%, EF = (LVVd-VVs)/LVVd× 100%.

All views were digitally stored in cine loops consisting of 300 frames. Subsequent analyses were performed off-line by an experienced sonographer who was blinded to the type of mouse model. LV volumes and ejection fraction (EF) were obtained using the standard 2D quantification software.

### Statistical analysis

SPSS 20 software was used for statistical processing. All data were expressed as mean ± standard deviation (Mean ± SD), two groups of data were analyzed by independent sample t test, and three or five groups of data were analyzed by one-way ANOVA. *P* < 0.05 means statistically significant difference.

## Result

### Quality control on human amnion mesenchymal stem cells

**Fig. 1:**
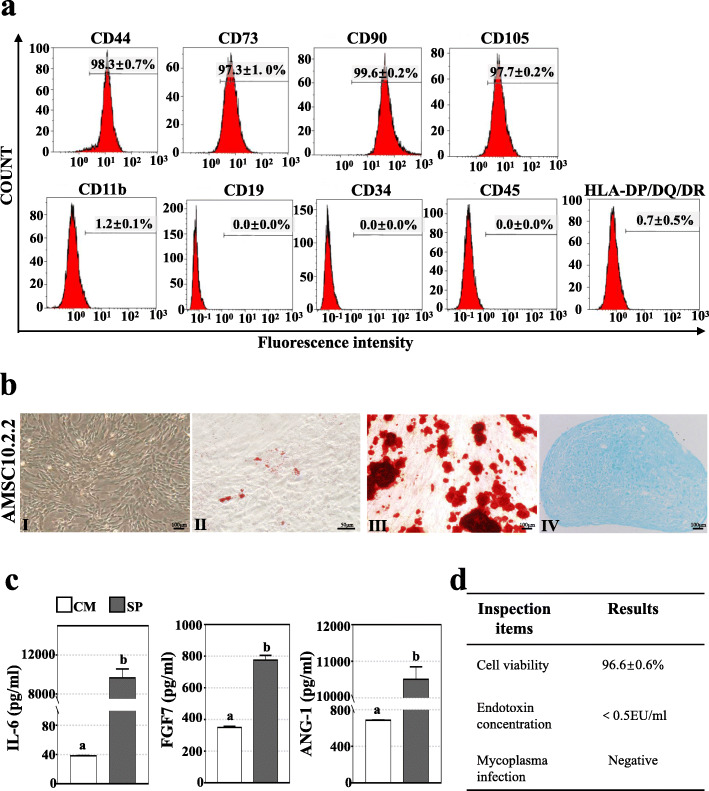
Quality control on hAMSCs. **a** The expression of MSC-specific surface makers were analyzed by flow cytometry to identify hAMSCs. Cells expressed high levels (> 95%) of CD73, CD44, CD105, and CD90, were negative, or expressed very low levels (< 2%) of CD11b, CD19, CD34, CD45, and HLA-DP/DQ/DR. **b** Cultured hAMSCs exhibited typical fibroblastic morphology (I) and could differentiate into adipocytes (II), osteoblasts (III), and chondrocytes (IV). **c** Concentration of secreting cytokines were evaluated by enzyme-linked immunosorbent assays in the cell culture supernatant (SP) and complete cell culture media (CM). Significant high concentrations of IL-6, FGF7, and ANG-1 were detected in culture supernatant (SP). **d** hAMSCs viability, bacteria contamination, and mycoplasma infection were tested by living cell counting, endotoxin detection, and real-time fluorescence quantitative polymerase chain reaction, respectively. hAMSCs showed a high viability (96.6 ± 0.6%), negative bacteria contamination and mycoplasma infection. Experiments were repeated at least for 3 times. Error bars indicate SEM. Different lowercase letters represent the significantly statistical differences (*P* < 0.05)

The fifth-passage hAMSCs from identified cell line of AMSC10 were freshly prepared, and release inspection on cell quality was carried before transplantation. The expression levels of mesenchymal stem cell (MSC)-specific surface molecular markers were evaluated and cytokines in cell culture supernatant were detected to identify and assess biological activity of hAMSCs respectively. Our result showed a high expression of CD44 (98.33 ± 0.66%), CD73 (97.27 ± 0.97%), D90 (98.58 ± 0.18%), CD105 (97.68 ± 0.17%), and a no or very low expression of CD11b (1.21 ± 0.06%), CD19 (0.00 ± 0.00%), CD34 (0.00 ± 0.00%), CD45 (0.00 ± 0.00%), and HLA-DP/DQ/DR (0.66 ± 0.53%) (Fig. 1a). Consistent with other types of MSCs, the hAMSCs resembled morphology of fibroblasts and had the ability to differentiate into osteoblasts, osteoblasts, and chondrocytes (Fig. 1b). Also, high concentrations of secreting cytokines of IL-6 (9655 ± 917 pg/mL), FGF7 (775.1 ± 30.01 pg/mL), and ANG-1 (10505 ± 344.3 pg/mL) were detected in cell culture supernatant (Fig. 1c). Meanwhile, prepared hAMSCs showed a high viability (96.6 ± 0.6%) and negative bacteria contamination and mycoplasma infection (Fig. 1d). Above data indicated a high quality of prepared hAMSCs.

### Establishment of age-related diminished ovarian reserve mouse model

**Fig. 2:**
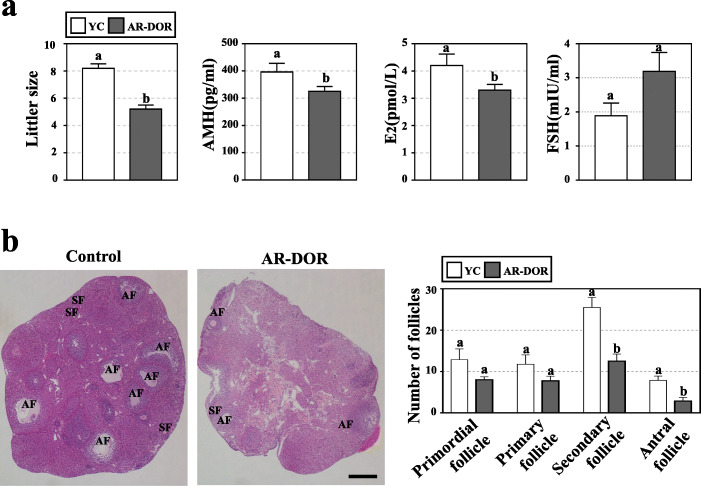
Establishment of age-related diminished ovarian reserve (AR-DOR) mouse model. **a** The litter size and serum level of hormones (by ELISA kit) were analyzed to access ovarian function. The litter size and levels of AMH and E2 were significantly decreased in AR-DOR mice and the level of FSH was increased in AR-DOR mice. **b** Histological analysis of paraffin-embedded sections and hematoxylin and eosin (HE) staining on mouse ovaries. The number of secondary and antral follicles was significantly decreased in AR-DOR mice. AMH: anti-Mullerian hormone; E2: estradiol; FSH: follicle stimulating hormone. AF: antral follicle; SF: secondary follicle. Scale bar 500 μm. *n* = 8. Error bars indicate SEM. Different lowercase letters represent significantly statistical differences (*P* < 0.05)

According to previous report, 32-week-old C57BL/6 mice are equivalent to about 35 years old of human [32], whose fertility has been proved gradually decreasing [33]. Here, the 32-week-old mice were selected to establish the age-related diminished ovarian reserve (AR-DOR) model. And 12-week-old female mice were set as young control (YC). The averaged littler size of the AR-DOR mice was significantly less than that of YC mice (8.2 ± 1.0 vs 5.4 ± 1.4, *P* < 0.05) (Fig. 2a). The AR-DOR mice also showed significantly decreased levels of AMH (394.6 ± 90.2 pg/mL vs 325.3 ± 50.9 pg/mL, *P* < 0.05) and E2 (4.2 ± 1.2 pmol/L vs 3.3 ± 0.6 pmol/L, *P* < 0.05), while they had an increased level of FSH (1.9 ± 0.4 mIU/mL vs 3.2 ± 0.6 mIU/mL, *P* > 0.05) in serum compared to the YC mice (Fig. 2a). Follicle counting results showed that the selected AR-DOR mice with a decreased number of primordial (8.3 ± 2.1 vs 12.9 ± 7.4, *P* > 0.05), primary (8.1 ± 3.2 vs 11.8 ± 6.5, *P* > 0.05), secondary (12.9 ± 5.2 vs 25.5 ± 6.9, *P* < 0.05), and antral (3.1 ± 2.5 vs 7.9 ± 2.9, *P* < 0.05) follicles compare to YC mice (Fig. 2b). Thus, age-related diminished ovarian reserve (AR-DOR) mouse model was established, with significantly less litter size, lower levels of AMH and E2 in serum, and smaller number of follicles in ovary (Fig. 2).

### hAMSC transplantation and in vivo tracing

**Fig. 3:**
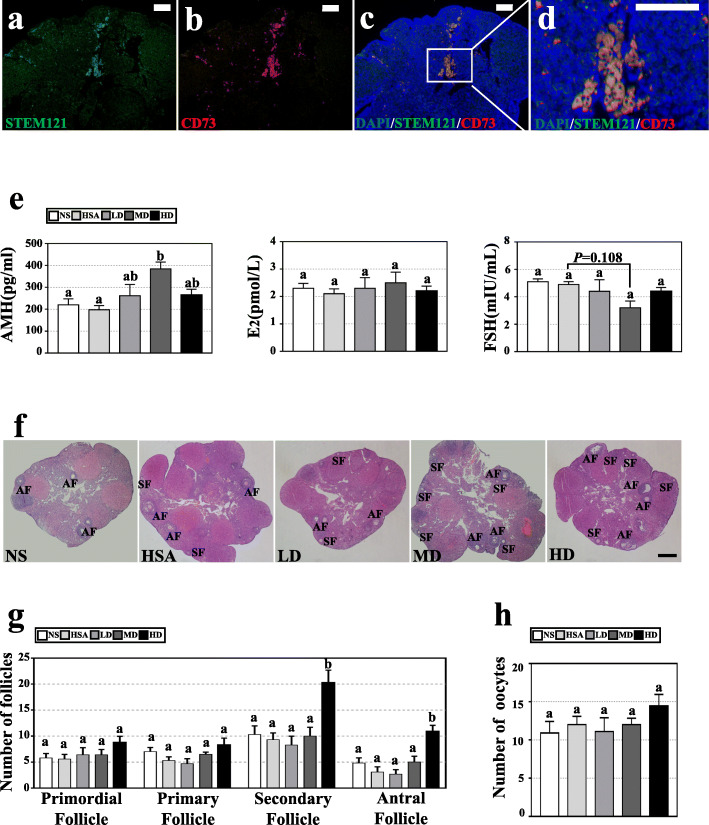
hAMSCs were homing in ovary and improved reproductive function after transplantation. **a–d** hAMSC homing and differentiation were traced by immunofluorescence experiments. Human-specific antibody of STEM121 (green signal) and mesenchymal stem cell-specific surface marker of CD73 (red signal) co-localization in the ovary. The sections were from middle dose of hAMSCs transplanted in ovary. Blue signal: 4,6-diamidino-2-phenyiindole (DAPI) staining. Scale bar 100 μm. **e** Serum levels of sex hormone were analyzed by ELISA kit. The AMH level was significantly increased and the FSH level was significantly decreased in the MD group. **f** Histological analysis of hematoxylin and eosin (HE) staining was performed to observe ovary. There were more follicles in the MD and HD groups. Scale bar, 500 μm. **g** Follicle counting showed the number of primordial and primary follicles in the HD group, and secondary and antral follicles in the MD and HD groups were significantly increased. **h** Retrieval oocyte counting indicated there was no significant difference in the numbers of oocyte between different groups. All above assays were performed at day 7 post-transplantation of hAMSCs. AMH: anti-Mullerian hormone; E2: estradiol; FSH: follicle stimulating hormone. NS: normal saline group; HSA: 1% human serum albumin group; LD: low-dose group; MD: middle-dose group; HD: high-dose group. *n* = 8. error bars indicate SEM. Different lowercase letters represent significantly statistical differences (*P* < 0.05)

In the study, relative low doses of hAMSCs were applied because AR-DOR mice are physically healthy. The AR-DOR mice were divided into 5 treatment groups and injected with the following preparations: normal saline (NS, control), 1% human serum albumin (HSA, resolver), low-dose (LD, 5.0 × 10^6^cells/kg body weight), middle dose (MD, 7.5 × 10^6^cells/kg body weight), and high dose (HD, 10.0 × 10^6^cells/kg body weight). Injections were continuously performed 3 times at a 4-day interval based on the following: (a) mouse has a 4–5-day estrous cycle [38] and (b) transplanted hAMSCs live in mouse at least for 1 week after tail vein injection (our unpublished data). All experimental mice lived to the planed endpoints and humanely sacrificed before next step analysis on tissues and organs, except that there were 4 mice dead from operation accident (1 in NS group, 1 in MD group and 2 in HD group). After injection, hAMSCs were detected in ovary (Fig. 3a–d) and all tested organs (Figure S1). Interestingly, except for ovary (resided in the mesenchyme) (Fig. 3a–d) and spleen (resided in the trabecular region) (Figure S1c, c’), the transplanted hAMSCs did not showed special residence in other tested organs (Figure S1). Meanwhile, co-expression human-specific gene STEM121 and MSC-specific surface marker of CD73 were observed in ovary (Fig. 3c, d). Our results showed that hAMSCs were homing in ovaries and at the same time maintained the characteristic of MSCs.

### hAMSC transplantation positively changed hormone levels in AR-DOR mice

The effect of hAMSC transplantation on AMH, E2, and FSH hormone secretion in the serum of treatment mice was investigated. The result showed that the AMH level of MD group (384.8 ± 87.0 pg/mL) was significantly increased compared to the NS (220.1 ± 78.3 pg/mL) and HSA groups (197.8 ± 54.0 pg/mL) (*P* < 0.05); meanwhile, AMH levels of LD (262.0 ± 144.5 pg/mL) and HD (265.2 ± 74.6 pg/mL) groups were obviously higher than those of NS and HSA groups (Fig. 3e). Additionally, the hAMSC transplantation groups all showed lower levels of the FSH, especially for the MD group (2.5 ± 1.1 mIU/mL) though there was no statistical differences observed (*P* = 0.108). Similarly, though the E2 level of the MD group (2.5 ± 1.1 pg/mL) showed an upward trend compared to the other groups, the difference is not statistically significant (Fig. 3e). Besides, level of LH hormone in MD group (4.14 ± 3.68 mIU/mL) show an upward trend compared to the NS (2.13 ± 1.76 mIU/mL) and HSA (3.60 ± 1.30 mIU/mL) groups, but the difference is not statistically significant, too (Figure S2). These results demonstrated that hAMSC transplantation positively changed the sex hormone levels in serum in AR-DOR mice, with especially the middle dose (7.5 × 10^6^cells/kg body weight).

### hAMSC transplantation increased the number of follicles in AR-DOR mice

The effect of hAMSC transplantation on the number of follicles at different developmental stages was evaluated. Fundamentally, the HD group showed the highest number of secondary (20.3 ± 6.7) and antral (10.9 ± 3.3) follicles and overwhelmed all the other groups (*P* < 0.05) (Fig. 3f, g); meanwhile, it showed an obvious increased number of primordial follicles (8.8 ± 3.3) and primary follicles (8.3 ± 3.7). Consistently, the most of oocytes were obtained from the HD group (14.4 ± 4.4) (Fig. 3h). However, there were no statistical differences between the other groups when follicle number (Fig. 3f, g) and oocyte number (Fig. 3 h) were compared. Our results demonstrated that a high dose (1.0 × 10^7^cells/kg body weight) of hAMSC transplantation promoted follicle development.

### hAMSC transplantation improved the quality of oocytes in AR-DOR mice

**Fig. 4:**
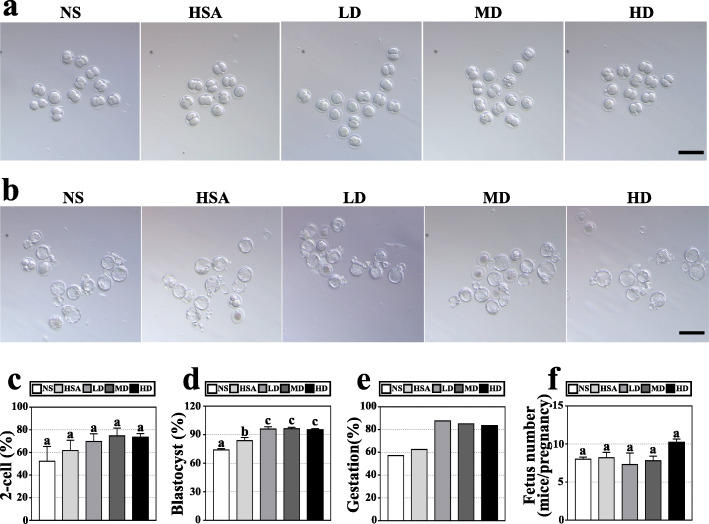
hAMSC transplantation improved oocyte quality and subsequent embryo development. **a** Morphology of 2-cell embryos. On the second day after fertilization, the zygote in the zona pellucida mitoses into 2 cells; no obvious difference was observed between groups under the microscope. Scale bar: 200 μm. **b** Morphology of blastocysts. On the fifth day after fertilization, the zygote mitosis develops into a blastocyst which forms a cyst cavity and/or breaking through the zona pellucida; no obvious difference was observed between groups under a microscope. Scale bar: 200 μm. **c** The rates of 2-cell formation (2-cell embryos/retrieval oocytes) were evaluated in groups and they were increased in the LD, MD, and HD groups. *n* = 8. **d** Rates of blastocyst formation (blastocyst embryos/2-cell embryos) were evaluated in groups and they were significantly increased in the LD, MD, and HD groups. *n* = 8. **e** The rates of gestation (pregnant mice with live fetus/total mice) were evaluated in groups and they were increased in the LD, MD, and HD groups. **f** Numbers of average live fetus were evaluated in groups. The HD group had a significantly higher number of live fetus. The number of mice analyzed in **e** and **f** was 7, 8, 8, 7, and 6, respectively for NS, HSA, LD, MD, and HD groups. Assays on 2-cell formation (**a**, **c**) and blastocyst formation (**b**, **d**) were performed at day 7 post-transplantation of hAMSCs. Assays on gestation (**e**) and live fetus (**f**) were performed at about day 15 post-transplantation of hAMSCs. NS: normal saline group; HSA: 1% human serum albumin group; LD: low-dose group; MD: middle-dose group; HD: high-dose group. Error bars indicate SEM. Different lowercase letters represent significantly statistical differences (*P* < 0.05)

The effects of hAMSC transplantation on the quality of oocyte were evaluated through observing the rates of 2-cell formation (2-cell embryos/retrieval oocytes), blastocyst formation (blastocyst embryos/2-cell embryos) and gestation (pregnant mice with alive fetus/total mice), and the average alive fetus numbers in every pregnant mouse. The results showed that all dose groups had a higher 2-cell formation rate (LD 69.7 ± 19.4%; MD 74.7 ± 19.4%; HD 73.2 ± 9.9%) (Fig. 4a, c) and gestation rate (LD 87.50% (7/8); MD 85.71% (6/7); HD 83.33% (5/6)) compared to the NS (2-cell formation rate: 52.5 ± 37.0%; embryo gestation rate: 57.14% (4/7)) and HSA (2-cell formation rate: 52.5 ± 37.0%; gestation rate: 62.50% (5/8)) groups (Fig. 4e), though there was no statistical significance. Not surprisingly, significantly higher blastocyst formation rates were observed in the LD (95.9 ± 7.0%), MD (96.3 ± 4.0%), and HD (95.1 ± 3.5%) groups compared to the NS (73.9 ± 4.2%) (*P* < 0.05) and HSA (83.8 ± 9.1%) groups (*P* < 0.05) (Fig. 4b, d). Similarly, the HD group (10.2 ± 1.3) presented obviously higher average number of live fetus than the NS (8.0 ± 0.8) and HSA (8.2 ± 2.0) groups though statistical differences were not observed, while there was no obvious difference between other groups (Fig. 4f). Our results demonstrated that hAMSC transplantation enhanced the quality of oocyte and subsequent embryo development in AR-DOR mice, especially in the HD group.

### hAMSC transplantation improved the status of ovarian cells in AR-DOR mice

**Fig. 5:**
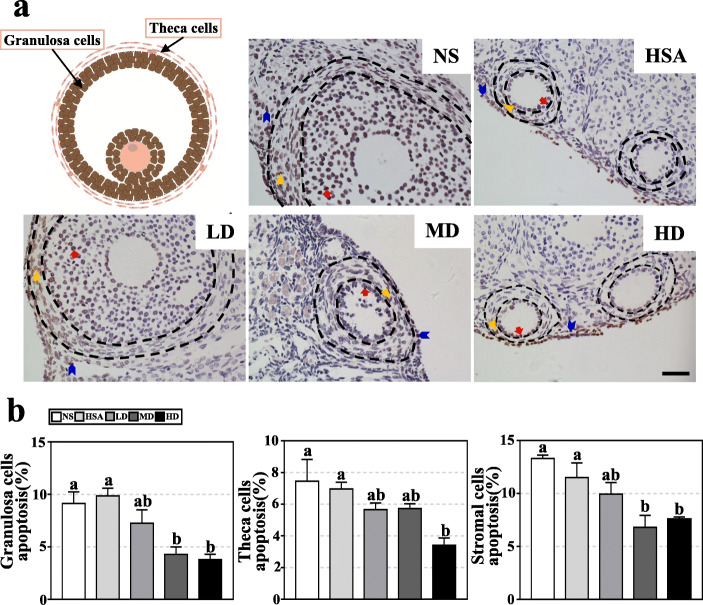
hAMSC transplantation repressed apoptosis of ovarian cells in AR-DOR mice. **a** Histological analysis on mouse ovary was carried out by TUNEL assay and microscope observation. Less apoptotic granulosa and stromal cells were observed in hAMSC-transplanted groups (LD, MD, and HD). Triangles indicate apoptotic granulosa cells. Arrows indicate apoptotic stromal cells. **b** Apoptosis rates of granulosa cells, theca cells, and stromal cells were calculated in groups. The rates of apoptosis in granulosa and stromal cells were significantly decreased in the MD and HD groups. The rate of apoptosis in theca cells was significantly decreased in the HD group. NS: normal saline group; HSA: 1% human serum albumin group; LD: low-dose group; MD; middle-dose group; HD: high-dose group. Scale bar 100 μm. *n* = 8. Error bars indicate SEM. Different lowercase letters represent significantly statistical differences (*P* < 0.05)

Apoptosis of supporting cells in ovary tightly correlates with oocyte quality and subsequent embryo development [39]. The effects of hAMSC transplantation on the apoptosis level in granulosa, theca, and stromal cells were estimated. Compared to the NS group (9.13 ± 2.23%) and HSA group (9.86 ± 1.47%), the rates of apoptosis in granulosa cells were decreased in the LD (7.27 ± 2.21%), MD (4.28 ± 1.24%) (*P* < 0.05), and HD (3.79 ± 0.87%) (*P* < 0.05) groups (Fig. 5a, b). However, there was no significant difference between the LD, MD, and HD groups and between the NS, HSA, and LD groups (Fig. 5a, b). Compared to the NS (7.46 ± 2.74%) and HSA (6.97 ± 0.84%) groups, the rate of apoptosis in theca cells was significantly decreased in the HD group (3.40 ± 0.94%) (*P* < 0.05), and that in LD (5.65 ± 0.74%) and MD (5.72 ± 0.52%) groups also decreased, but no statistical differences were observed. The apoptosis levels in stromal cells indicated similar results as granulosa cells: MD (6.80 ± 1.99%) and HD (7.56 ± 0.50) groups were significantly lower than NS group (13.31 ± 0.55%) and HSA group (11.52 ± 2.77%); meanwhile, the apoptosis levels in the LD group was lower than the NS group and HSA group and higher than MD group and HD group, but the differences were not significant. These results indicated that the hAMSC transplantation reduced the apoptosis level of ovarian cells in AR-DOR mice, in a dose-dependent manner.

In addition, the expression of Cyp17a1 (which is a key enzyme for androgen synthesis in follicular thecal cells) in hAMSC group showed an upward trend compared to the NS and HSA groups. And the expression of FSHR located in the granulosa cell surface showed the same trend. These results indicated that the hAMSC transplantation could increase the expression of protein involved in reproductive hormone signaling in ovarian cells to improve the ovarian function (Figure S3).

### hAMSC transplantation enhanced Ampk/FoxO3a signaling pathway in the ovary of AR-DOR mice

**Fig. 6:**
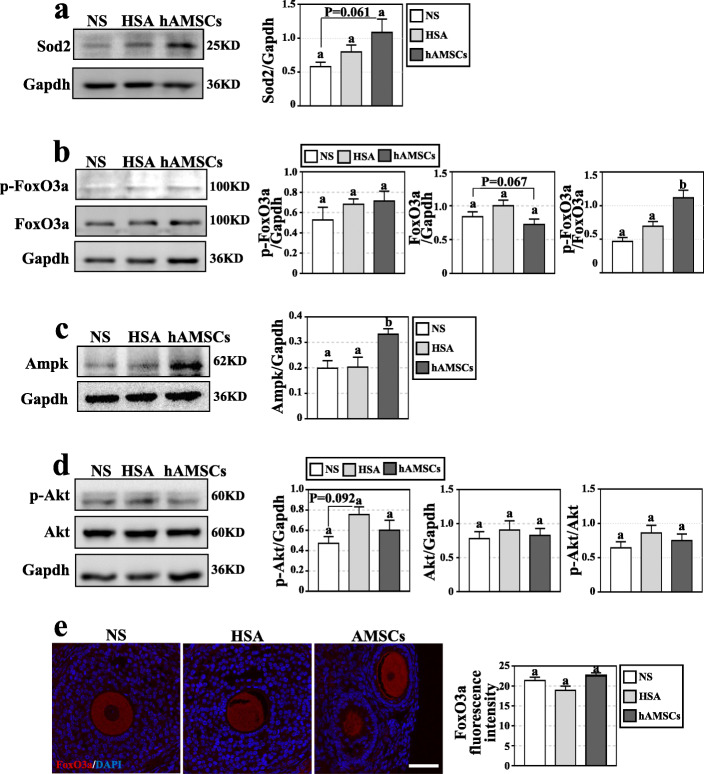
hAMSC transplantation enhanced oxidation resistance and promoted Ampk/Foxo3a signaling in ovary of AR-DOR mice. **a-d** Expression levels of proteins in mouse ovary were determined by Western blot analysis. **a** The expression of antioxidative Sod2 was increased in the hAMSC group. **b** The expression level of p-FoxO3a and FoxO3a in the hAMSC group was similar to that of NS and HSA groups, but ratio of p-FoxO3a/FoxO3a was significantly improved in the hAMSC group. **c** The expression of Ampk was significantly promoted in the hAMSC group. **d** No obvious difference of p-Akt and Akt expression was observed between groups. **e** Expression of FoxO3a in oocyte were determined by immunofluorescence experiments. stronger signals were observed in the hAMSC group. NS: normal saline group; HSA: 1% human serum albumin group; hAMSCs: hAMSC transplantation group (7.5×10^6^ cells/kg body weight). Scale bar 100 μm. *n* = 6. Error bars indicate SEM. Different lowercase letters represent significantly statistical differences (*P* < 0.05)

Aging ovaries are associated with an imbalanced redox state which increases reducing reactive oxygen species (ROS) relative to antioxidant signaling [40]. It is reported that FoxO3a is crucial for oocyte maturation and subsequent embryonic development through activating mitophagy, reducing ROS-induced mitochondrial injury [41, 42]. Here we tested if FoxO3a signaling participated in the ovarian function improvement after hAMSC transplantation. We found that the expression of superoxide dismutase 2 (Sod2) (Fig. 6a) and the ratio of phosphorylated FoxO3a (p-FoxO3a) to total FoxO3a (*P* < 0.05) (Fig. 6b) was increased. Consistently, the enhanced expression of FoxO3a in cytoplasm was observed (Fig. 6e). Additionally, expression level of Ampk, the catalyzer promoting FoxO3a phosphorylation, increased as well (*P* < 0.05) (Fig. 6c). Interestingly, expression of Akt and phosphorylated Akt were not changed (Fig. 6d). Our results indicated that the oxidation resistance and Ampk/FoxO3a signaling pathway were both enhanced in the ovaries of AR-DOR mice after hAMSC transplantation.

## Discussion

There is a clear correlation between increasing maternal age and decreasing success in conceiving both spontaneously and after IVF [43, 44], which is attributed to the diminished ovarian reserve and decreased oocyte quality. Age is the independent factor which crucially affects IVF outcomes [45]. IVF cannot reverse the adverse impacts of advanced age (particularly in women over 40) though it can help part of younger women to have babies [46]. After IVF treatment, live birth rate is only 3.1% in women over 42 years old, while 46.8% in women under 35; the same trend parallels spontaneous conception rates [47]. Mature oocytes are developing from members of primordial follicle pool which is established in utero. Thus, depletion on primordial follicle pool and/or adverse microenvironment in the ovary can impede the maturing of oocyte [48–50]. Furthermore, aging-associated systems can also affect oocyte quality because the hypothalamus-pituitary-gonad (HPG) axis signaling synergistically regulates oocyte maturation [51].

Primordial follicle pool is declining irreversibly until menopause, but exceeding activation of primordial follicles asynchronous with age can result in POF; for example, almost all of young women with cancer suffered POF after receiving radiation and chemotherapy [52–54]. The Pten/Akt/FoxO3a pathway is crucial in regulating quiescence or activation of primordial follicles [55–59]. The FoxO3a and phosphorylation FoxO3a are the critical factors, usually FoxO3a represses while phosphorylation FoxO3a activates the development of primordial follicles. Additionally, Fox3a is also involved in mitophagy in senescence oocytes [60]. Aging and many of the age-related diseases is tightly related to abnormal distribution and function of mitochondria [61]. Meanwhile, mitophagy is a major process to maintain normal quality and quantity of mitochondria in cells, especially in oocytes because they contain a large number of mitochondria to meet the demand of energy production during oocyte maturation and subsequent embryonic development [62]. There are also reports that demonstrate that AMPK is the guardian of metabolism and mitochondrial homeostasis, crucial in the regulation of metabolism via AMPK/TSC/mTOR pathway and of mitophagy through AMPK/FoxO3a signaling [63].

In the study, an AR-DOR mouse model with ovarian function equivalent to that of 35 years old women [32] was established. Our results showed hAMSC injection significantly improve the ovarian function of AR-DOR mice, while the expression of Ampk and the ratio of phosphorylation FoxO3a to FoxO3a increased (Fig. 6b, c), proposing that hAMSC transplantation might improve ovarian function through enhancing mitophagy in oocyte. Furthermore, increased phosphorylation FoxO3a might simultaneously promote the activation of primordial follicles because the HD group had higher numbers of follicles at all of the four development stages (especially for the secondary and antral follicles) in AR-DOR mice (Fig. 3f, g). Aging can result in excess accumulation of ROS in ovary and impair oocyte maturation [12, 13]. hAMSC transplantation increased expression of antioxidative gene of Sod2 (Fig. 6a) while it repressed apoptosis in granulosa, theca, and stromal cells (Fig. 5), demonstrating that hAMSCs might also promote oocyte maturation by improving ovarian microenvironment, consistent with previous reports [24, 25, 29, 30]. Besides, serum hormone levels of AR-DOR mice were somehow rebalanced (Fig. 3e), implying that the HPG axis signaling was regulated too after hAMSC transplantation. Interestingly, hAMSCs resided in ovaries but still presented the features of MSCs (Fig. 3c, d), meaning that they functioned by secreting cytokines [64] (Fig. 1b) and/or vesicles [24, 29] rather than differentiation into tissue-specific cells. Those precise hAMSC-secreting components effective in ovarian function repairability and the function mechanism would be explored in future research.

It is reported that immune response arising from heterografts [65] may produce many inflammatory factors which may affect ovarian function [66, 67]. In our study, HFF transplantation could improve ovarian function of DOR mice to a certain extent, including changing serum hormone levels (Figure S4a) and promoting follicle development (Figure S4b-d) and embryonic development (Figure S5). However, there was no statistically significant difference between the HFF and HSA groups. Meanwhile, the hAMSC group showed an obviously higher serum level of AMH and blastocyst formation rate than the HSA group (*p* < 0.05 and *p* < 0.05, respectively) and HFF group (*p* = 0.07 and *p* = 0.10, respectively) (Figures S4, S5). Previous reports suggest that HFFs possess some of MSCs’ characteristics and function in regeneration or reparation [68, 69], and our FASC results really indicated that HFFs expressed a high level of specific cell surface markers of MSCs (Figure S6). It means that HFFs may have some positive effects on ovary function of DOR mice, and more experimental animals should be employed to confirm the superior effects of hAMSCs, though our present data based on a small number of experimental animals, however, clearly suggests that hAMSCs improve ovarian function of DOR mice attribute to specific activity of cells rather than common immune reactions after heterograft.

In our study, 300 μl fluid was injected into each experimental mouse in a short time. Analysis on heart function was performed to assess if the relative higher injection volume may result in congestive heart failure. Our results showed that all the three groups of mice had normal M-mode echocardiography (Figure S7a), and normal heart rates (HR), ejection fraction (EF), and fractional shortening (FS); meanwhile, all other measured parameters of heart function were similar (Figure S7b). Besides, there was no obvious difference observed in pathological sections of hear tissues between groups (Figure S7c). Our data suggested that 300 μl fluid injection had no obvious negative effects on the heart of transplanted mice.

More attention should be paid to the dose in cell therapy because in our study a relatively small difference (about 30%) in dosage had shown significant difference on therapeutic effects (Figs. 3 and 4). In view of the size, potential immunogenicity of transplanted cells, and they would live in a recipient body for a relatively long time, an effective and safe dose as low as possible is recommended in future clinical application.

Furthermore, ovarian function extremely damaged would not be recovered by hAMSC transplantation. We tested the limitation of cell therapy by an X-ray-induced POF mouse model. The ovaries of POF mice were smaller and follicles were decreased significantly than that of young controls (Figure S8a), even no antral follicle was observed in the ovaries (Figure S8b). After hAMSC transplantation, though the serum levels of AMH, E2, and FSH were changed significantly (Figure S8c), in ovary severe fibrillation was ameliorated (Figure S8e) and primordial follicles were activated, yet no antral follicle was detected in POF mice (Figure S8d). Our results suggest that in future clinical application, ovarian function of patients should be carefully evaluated before cell transplantation.

It is reported that several types of MSCs (PD-MSCs, UCMSCs, ADSCs) or their secretions are effective in improving ovarian function [17–25]. As a member of MSC family, if hAMSCs showed any difference from other type of MSCs? And if hAMSCs have advantages in AR-DOR treatment? During the development of the embryo, the placental tissue originates before gastrulation, meaning that some cells from this tissue may retain multipotent/pluripotent characteristics [70]. This distinguishes placental tissue (amnion)-derived hAMSCs from MSCs originated from other adult human tissues. Of reported MSCs, the origin of hUCMSCs is most close to that of hAMSCs. Interestingly, our results of protein microarray showed hAMSCs and hUCMSCs (derived from the same donor) had different cytokine pool in the cell culture supernatant (unpublished data). Several cytokines closely related to reproductive development show a higher expression level in the supernatant of culture hAMSCs, including FGF7, LIF, and VEGF (Table S1). We speculate that hAMSCs may be more powerful in treatment of AR-DOR compared to other type of MSCs. However, in vitro and in vivo experiments should be performed to testify to the speculation in future study.

## Conclusion

hAMSC transplantation via tail vein promotes follicle activation, oocyte maturation, and subsequent embryonic development in AR-DOR mice. The transplanted hAMSCs are homing in ovary while maintaining the characterization of MSCs, effecting by cell secretion to activated AMPK/FoxO3a signaling and downstream antioxidation pathway. Although the underlying mechanism is unclear yet, hAMSC transplantation might promise a prospect candidate treatment for AR-DOR therapy.

## Supplementary Information

**Additional file 1: Figure S1.** Tracing of hAMSCs *in vivo.* hAMSCs homing was traced in different organs by immunofluorescence experiments with human specific antibody of STEM121 (green signal). hAMSCs were detected in all analyzed organs, including lung (a, a'), thymus (b, b'), spleen (c, c'), heart (d, d'), kidney (e, e') and liver (f, f'). Scar bar: 100μm.

**Additional file 2: Figure S2.** hAMSCs transplantation improved slightly the level of LH hormone. Serum level of LH hormone was analyzed by ELISA Kit. The LH level in hAMSCs group showed an upward trend, but the difference was not statistically significant.

**Additional file 3: Figure S3.** hAMSCs transplantation improved slightly the expression of FSHR and Cyp17a1. Cyp17a1 is a key enzyme for androgen synthesis in follicular theca cells, and FSHR is a granulosa specific cell surface protein which stimulated by the hormone FSH to promote follicular growth. The expression of FSHR and Cyp17a1 in hAMSCs group showed an upward trend, but the differences were not statistically significant.

**Additional file 4: Figure S4.** hAMSCs showed superior effects in improving reproductive function after transplantation. a Serum levels of sex hormone were analyzed by ELISA kit. The AMH level was significantly increased in hAMSCs group. b Histological analysis of hematoxylin and eosin (HE) staining was performed to observe ovary. There were more follicles in hAMSCs group. Scar bar, 500μm. c Follicle counting showed number of secondary follicles in hAMSCs group were significantly increased. d Retrieval oocyte counting in hAMSCs group had increased trend compared to other groups.

**Additional file 5: Figure S5.** hAMSCs showed superior effects in improving oocyte quality after transplantation. a Morphology of 2-cell embryos and the rate of 2-cell formation. The rate of 2-cell formation was increased in hAMSCs group. Scar bar: 200μm. b Morphology of blastocysts and the rate of blastocyst formation. The rate of blastocyst formation was significantly increased in hAMSCs group. Scar bar: 200μm.

**Additional file 6: Figure S6.** HFFs expressed high level of specific cell surface markers of MSCs. The expression of MSCs specific surface makers were analyzed by flow cytometry to identify hAMSCs. Cells expressed high level (>95%) of CD73, CD44, CD105, CD90, were negative or expressed very low level (<2%) of CD11b, CD19, CD34, CD45 and HLA-DP/DQ/DR.

**Additional file 7: Figure S7.** Assessment of cardiac function after injection. a Representative images showing M mode echocardiography of mice. b Echocardiographic results of the heart rates (HR), ejection fraction (EF), fractional shortening (FS), cardiac output (CO), left ventricular dimension in systole (LVDs), left ventricular dimension in diastole (LDd), left ventricular volume in systole (LVVs), left ventricular volume in diastole (LVVd), left ventricular posterior wall thickness in systole (LVPWs), left ventricular posterior wall thickness in diastole (LVPWd), left ventricular anterior wall thickness in systole (LVAWs), left ventricular anterior wall thickness in diastole (LVAWd). There were no obvious differences of echocardiographic results between groups. c Histological analysis of hematoxylin and eosin (HE) staining was performed to observe heart micro structure. There was no obvious difference was observed between groups under pathological analysis. Scar bar, 200μm. NI, no injection group; HSA, human serum albumin injection group; hAMSCs: hAMSCs injection group.

**Additional file 8: Figure S8.** hAMSCs transplantation limitedly improved ovarian functioning in X-ray induced premature ovarian failure (DOF)model mice. a Histological analysis on ovaries of young control group (YC) and X-ray induced POF group (POF). The POF group showed smaller ovary and less follicles than YC group. Scar bar: 500μm. b Follicle counting in the YC and POF group, all but number of primary follicles in POF group were significantly decreased. c Serum levels of sex hormones were evaluated by ELISA kit. Levels of AMH and E2 were significantly decreased by X-ray treatment (POF+NS group) but increased significantly (POF+hAMSCs group) after hAMSCs transplantation, and Levels of FSH showed inverse responses. d Histological analysis on ovaries in different treatment groups. hAMSCs transplantation promoted follicle activation (POF+hAMSCs group). Yellow dot-circles indicated early stage of follicles. Scar bar: 500μm. e Fibrosis in ovaries was evaluated by observation under transmission electron microscope. hAMSCs transplantation alleviated fibrosis (POF+hAMSCs group). Scar bar: 2μm. All ovarian sections were stained with hematoxylin and eosin (HE). AF: antral follicle; SF: secondary follicle. POF+NS: POF plus normal saline injection group; POF+hAMSC: POF plus hAMSCs injection group. AMH: anti-Mullerian hormone; E2: estradiol; FSH: follicle stimulating hormone. n=8. Error bars indicate SEM. Different lowercase letters represent the difference of expression levels that are significant (*P*<0.05).

**Additional file 9: Table S1. **Compared the relative expression of cytokines in culture supnantant of hAMSCs and hUMSCs.

## Data Availability

All data generated and/or analyzed during this study are included in this published article.

## Abbreviations

ANG-1: Angiopoietin-1
ADSCs: Adipose-derived stem cells
AFC: Antral follicle counting
AMH: Anti-Mullerian hormone
AR-DOR: Age-related Diminished Ovarian Reserve
ART: Artificial assisted reproduction
DOR: Diminished Ovarian Reserve
E2: Estradiol
ELISA: Enzyme-linked immunosorbent assay
FGF7: Fibroblast growth factor 7
FSH: Follicle-stimulating hormone
hAMSC: Human amnion-derived mesenchymal stem cell
HRP: Horseradish peroxidase
HSA: Human serum albumin
HuMenSCs: Human endometrial stem cells
IL-6: Interleukin-6
MSCs: Mesenchymal stem cells
NS: Normal saline
POF: Premature ovarian failure
PVDF: Polyvinyl fluoride
ROS: Reactive oxygen species
SDS-PAGE: Sodium dodecyl sulfate polyacrylamide gel electrophoresis
Sod2: Superoxide dismutase 2
UCMSCs: Human umbilical cord mesenchymal stem cells
