# Supplementary figures and images for: Human amnion-derived mesenchymal stem cells improved the reproductive function of age-related diminished ovarian reserve in mice through Ampk/FoxO3a signaling pathway
Source: Stem Cell Res Ther. 2021 Jun 2;12:317. doi: 10.1186/s13287-021-02382-x (PMC8173966; doi:10.1186/s13287-021-02382-x)

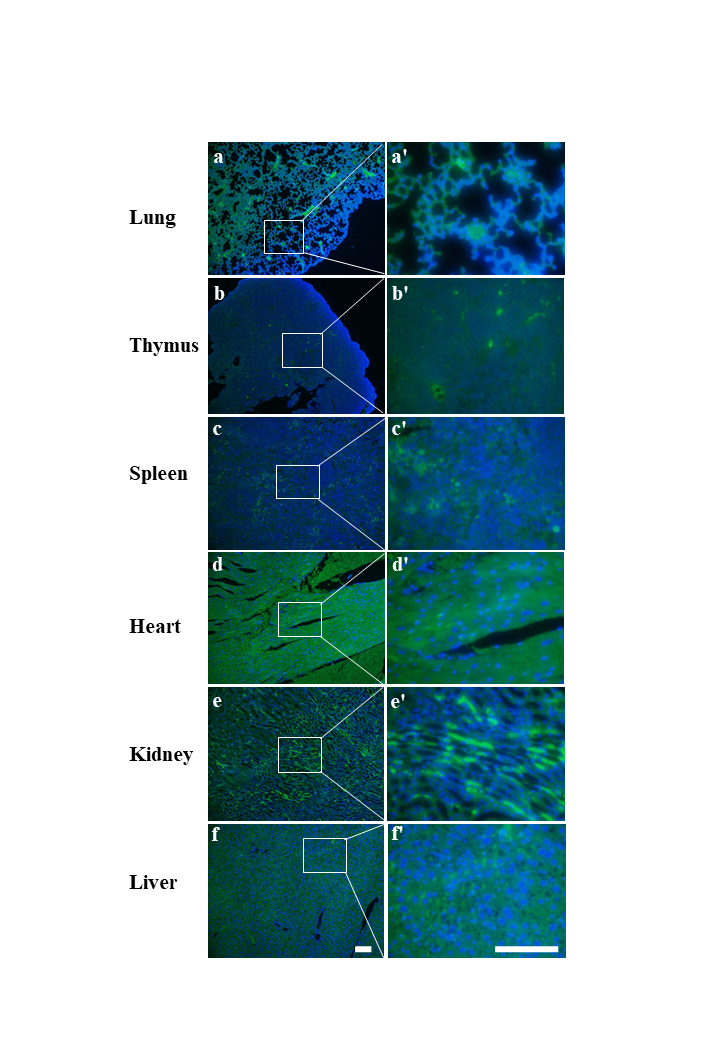

Supplement: Supplementary file 1 — Additional file 1: Figure S1. Tracing of hAMSCs in vivo. hAMSCs homing was traced in different organs by immunofluorescence experiments with human specific antibody of STEM121 (green signal). hAMSCs were detected in all analyzed organs, including lung (a, a'), thymus (b, b'), spleen (c, c'), heart (d, d'), kidney (e, e') and liver (f, f'). Scar bar: 100μm. [file 13287_2021_2382_MOESM1_ESM.tif]

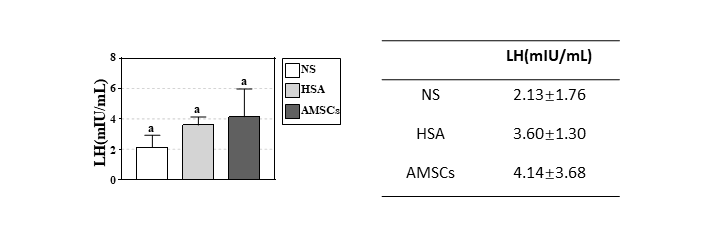

Supplement: Supplementary file 2 — Additional file 2: Figure S2. hAMSCs transplantation improved slightly the level of LH hormone. Serum level of LH hormone was analyzed by ELISA Kit. The LH level in hAMSCs group showed an upward trend, but the difference was not statistically significant. [file 13287_2021_2382_MOESM2_ESM.tif]

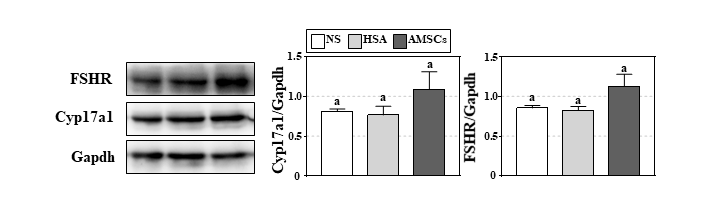

Supplement: Supplementary file 3 — Additional file 3: Figure S3. hAMSCs transplantation improved slightly the expression of FSHR and Cyp17a1. Cyp17a1 is a key enzyme for androgen synthesis in follicular theca cells, and FSHR is a granulosa specific cell surface protein which stimulated by the hormone FSH to promote follicular growth. The expression of FSHR and Cyp17a1 in hAMSCs group showed an upward trend, but the differences were not statistically significant. [file 13287_2021_2382_MOESM3_ESM.tif]

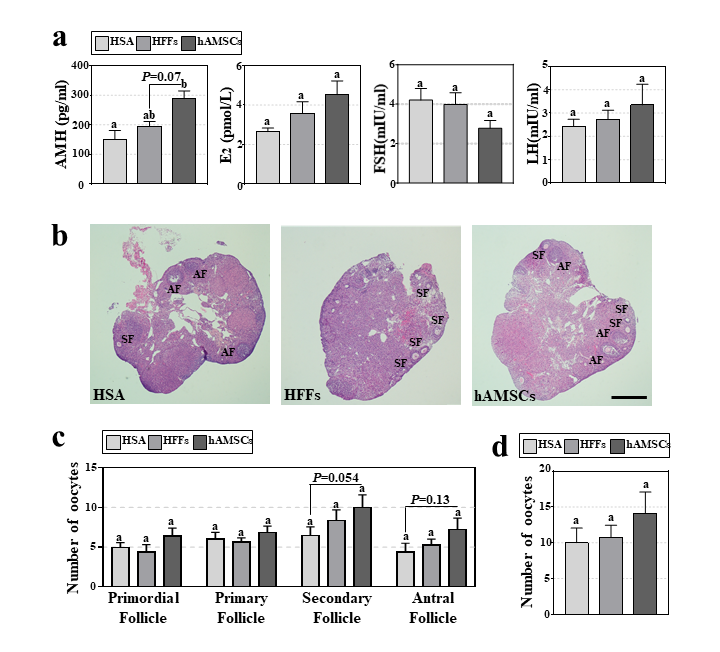

Supplement: Supplementary file 4 — Additional file 4: Figure S4. hAMSCs showed superior effects in improving reproductive function after transplantation. a Serum levels of sex hormone were analyzed by ELISA kit. The AMH level was significantly increased in hAMSCs group. b Histological analysis of hematoxylin and eosin (HE) staining was performed to observe ovary. There were more follicles in hAMSCs group. Scar bar, 500μm. c Follicle counting showed number of secondary follicles in hAMSCs group were significantly increased. d Retrieval oocyte counting in hAMSCs group had increased trend compared to other groups. [file 13287_2021_2382_MOESM4_ESM.tif]

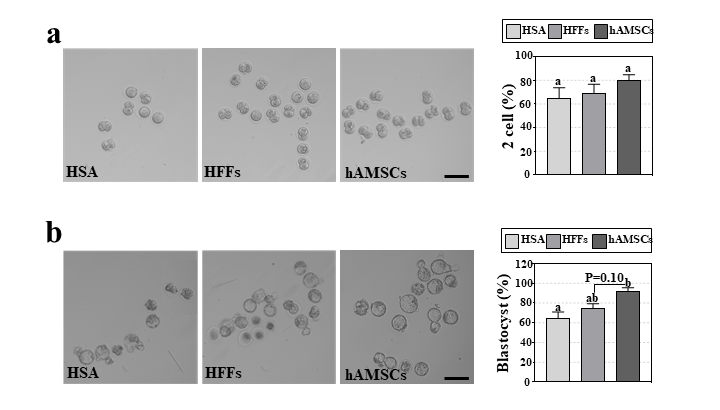

Supplement: Supplementary file 5 — Additional file 5: Figure S5. hAMSCs showed superior effects in improving oocyte quality after transplantation. a Morphology of 2-cell embryos and the rate of 2-cell formation. The rate of 2-cell formation was increased in hAMSCs group. Scar bar: 200μm. b Morphology of blastocysts and the rate of blastocyst formation. The rate of blastocyst formation was significantly increased in hAMSCs group. Scar bar: 200μm. [file 13287_2021_2382_MOESM5_ESM.tif]

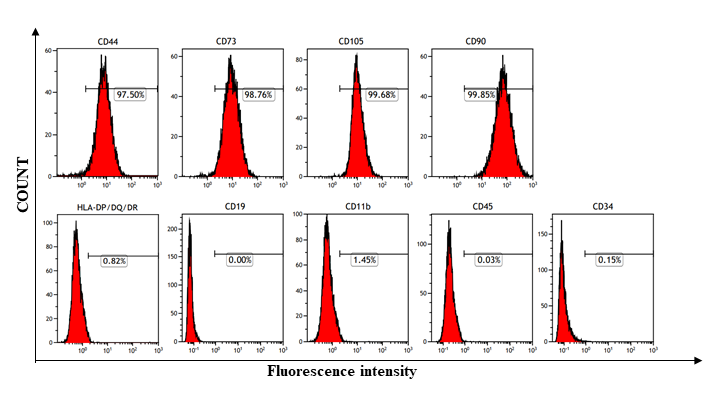

Supplement: Supplementary file 6 — Additional file 6: Figure S6. HFFs expressed high level of specific cell surface markers of MSCs. The expression of MSCs specific surface makers were analyzed by flow cytometry to identify hAMSCs. Cells expressed high level (>95%) of CD73, CD44, CD105, CD90, were negative or expressed very low level (<2%) of CD11b, CD19, CD34, CD45 and HLA-DP/DQ/DR. [file 13287_2021_2382_MOESM6_ESM.tif]

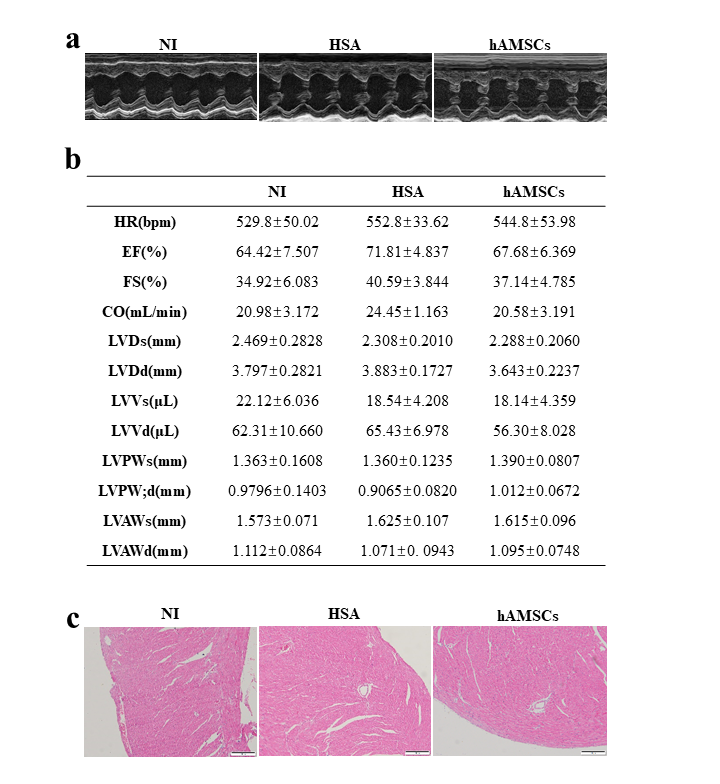

Supplement: Supplementary file 7 — Additional file 7: Figure S7. Assessment of cardiac function after injection. a Representative images showing M mode echocardiography of mice. b Echocardiographic results of the heart rates (HR), ejection fraction (EF), fractional shortening (FS), cardiac output (CO), left ventricular dimension in systole (LVDs), left ventricular dimension in diastole (LDd), left ventricular volume in systole (LVVs), left ventricular volume in diastole (LVVd), left ventricular posterior wall thickness in systole (LVPWs), left ventricular posterior wall thickness in diastole (LVPWd), left ventricular anterior wall thickness in systole (LVAWs), left ventricular anterior wall thickness in diastole (LVAWd). There were no obvious differences of echocardiographic results between groups. c Histological analysis of hematoxylin and eosin (HE) staining was performed to observe heart micro structure. There was no obvious difference was observed between groups under pathological analysis. Scar bar, 200μm. NI, no injection group; HSA, human serum albumin injection group; hAMSCs: hAMSCs injection group. [file 13287_2021_2382_MOESM7_ESM.tif]

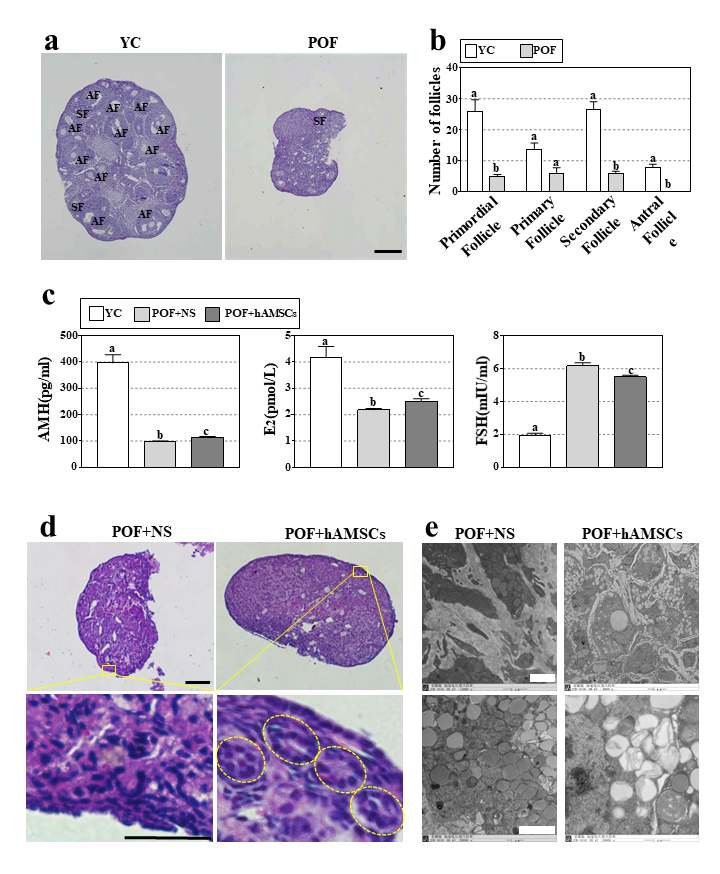

Supplement: Supplementary file 8 — Additional file 8: Figure S8. hAMSCs transplantation limitedly improved ovarian functioning in X-ray induced premature ovarian failure (DOF)model mice. a Histological analysis on ovaries of young control group (YC) and X-ray induced POF group (POF). The POF group showed smaller ovary and less follicles than YC group. Scar bar: 500μm. b Follicle counting in the YC and POF group, all but number of primary follicles in POF group were significantly decreased. c Serum levels of sex hormones were evaluated by ELISA kit. Levels of AMH and E2 were significantly decreased by X-ray treatment (POF+NS group) but increased significantly (POF+hAMSCs group) after hAMSCs transplantation, and Levels of FSH showed inverse responses. d Histological analysis on ovaries in different treatment groups. hAMSCs transplantation promoted follicle activation (POF+hAMSCs group). Yellow dot-circles indicated early stage of follicles. Scar bar: 500μm. e Fibrosis in ovaries was evaluated by observation under transmission electron microscope. hAMSCs transplantation alleviated fibrosis (POF+hAMSCs group). Scar bar: 2μm. All ovarian sections were stained with hematoxylin and eosin (HE). AF: antral follicle; SF: secondary follicle. POF+NS: POF plus normal saline injection group; POF+hAMSC: POF plus hAMSCs injection group. AMH: anti-Mullerian hormone; E2: estradiol; FSH: follicle stimulating hormone. n=8. Error bars indicate SEM. Different lowercase letters represent the difference of expression levels that are significant (P<0.05). [file 13287_2021_2382_MOESM8_ESM.tif]
